# Microvascular changes in macula and optic nerve head after femtosecond laser-assisted LASIK: an optical coherence tomography angiography study

**DOI:** 10.1186/s12886-020-01368-2

**Published:** 2020-03-17

**Authors:** Yan Zhang, Jianqing Lan, Dan Cao, Cheng Yang, Dawei Yang, Wenjuan Xie, Jin Zeng

**Affiliations:** Department of Ophthalmology, Guangdong Eye Institute, Guangdong Provincial People’s Hospital, Guangdong Academy of Medical Sciences, No. 106 Zhongshan Er Road, Guangzhou, 510080 China

## Abstract

**Background:**

To measure the microcirculation change of macula and optic nerve head before and after femtosecond laser assisted laser in situ keratomileusis.

**Methods:**

In total 45 eyes from 45 subjects, who underwent FS-LASIK during June 2017 to December 2017 in Guangdong Provincial People’s Hospital, were recruited in this study. Vessel density in macula and optic nerve head were measured by optical coherence tomography angiography before and after transient elevation in intraocular pressure caused by application of suction ring during surgery.

**Results:**

Vessel density (VD) at superficial (SCP) plexus of macular region did not differ after surgery (F(3,132) = 1.41, *P* = 0.24), while the deep (DCP) plexus of macular region significantly decreased 1 day after surgery (*P* = 0.001) but returned to its baseline value 1 month postoperatively (*P* = 0.1). Vessel density of optic nerve head region had no significant changes after surgery (F(2.51,95.18) = 0.6, *P* = 0.59).

**Conclusions:**

A short-term temporary decrease of vessel density at deep layer of macular region was observed in eyes undergoing FS-LASIK. However, the retinal capillary density went back to preoperative level 1 month after surgery. Therefore, transient IOP spike during FS-LASIK did not cause long-term decline of retinal microcirculation.

## Background

Femtosecond laser assisted laser in situ keratomileusis (LASIK) is currently one of the most commonly performed refractive surgery worldwide. During the surgery, a suction ring is applied on the anterior segments of the eye in order to immobilize the eyeball. IOP could be elevated to more than 90mmHg [1–3] in the vacuum affixation phase and return to normal after the suction ring was removed. Investigation revealed that sudden increase of IOP caused a temporary central retinal artery occlusion during the suction phase [4] and lead to a stop of retinal circulation. Potential ocular complications occur overtimes. Solitary cases of nonarteritic ischemic optic neuropathy (NAION) [5], iris atrophy [6], macular edema [7], macular hemorrhage [8] after LASIK were reported.

Previous study has investigated a temporary increase in blood flow at the lamina cribrosa region of the optical nerve head after surgery [9]. While the change of macular blood flow was currently unknown. Optical coherence tomography angiography (OCTA) is a new noninvasive technique that can visualize the retinal vascular network and quantify the vascular density in the macular area and optical nerve head (ONH). In this study, microcirculation in the macula and ONH were measured by OCTA to investigate the retinal blood change before and after the surgery.

## Methods

This prospective study was conducted from June 2017 to December 2017 at Guangdong Provincial People’s Hospital. A total of 48 patients were recruited in this study, one eye of each subject was randomly selected by a process of alternation. The research protocols were approved by the Institutional Review Broad at Guangdong Provincial People’s Hospital. The study followed the tenets of the Declaration of Helsinki and all subjects were thoroughly informed of the procedure and provided written informed consent.

The inclusion criteria for the surgery were as follows: patients were willing to receive the surgery and had good compliance and cognitive ability, age of 18 or older, stable refraction for at least 2 years, refractive error not greater than − 8.0 diopters (D) sphere or − 4.0D of astigmatism, no history of ocular surgery or any other ocular or systemic diseases. Eyes with possible keratoconus were excluded by using the keratoconus screening test of Pentacam HR(Oculus, Germany).

### Surgical procedures

All surgeries were performed by the same experienced surgeon (J.Z.). A suction ring was applied to the anterior segment of the eye in order to immobilize the eyeball, after which an 8.4 to 8.5 mm flap of 100 um thickness was cut with the femtosecond laser (IntraLase iFS 150, Abbott Medical Optics Inc., Santa Ana, California). The flap was separated and lifted, followed by ablation in a 6.0 to 6.2 mm optical zone using the Wavefront-guided excimer laser (Technolas 217z100 excimer laser platform, Bausch & Lomb, Rochester, NY). After repositioning the flap, postoperative topical medications were given.

### OCTA imaging

The RTVueXR Avanti device (V.2017.1.0.121; RTVueXR Avanti; Optovue, Inc., Fremont, CA, USA) is a new non-invasive imaging device utilizing SSADA (split-spectrum amplitude-decorrelation angiography) to detect vessels and blood flow through the motion provided by flowing erythrocytes. All patients received OCTA examination before and 1 day/ 1 week/ 1 month after surgery and all examinations were performed by the same ophthalmologist (Y.Z).

**Fig. 1:**
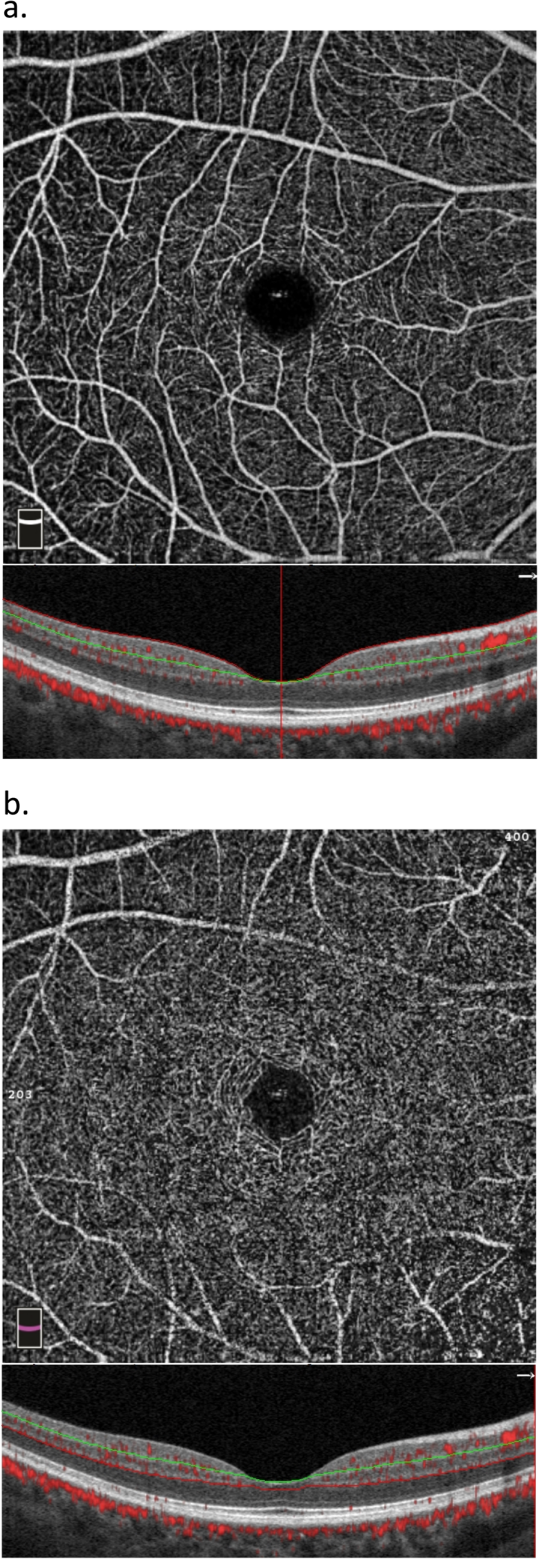
Example of vessel density image of superficial plexus (a) and deep plexus (b)

**Fig. 2:**
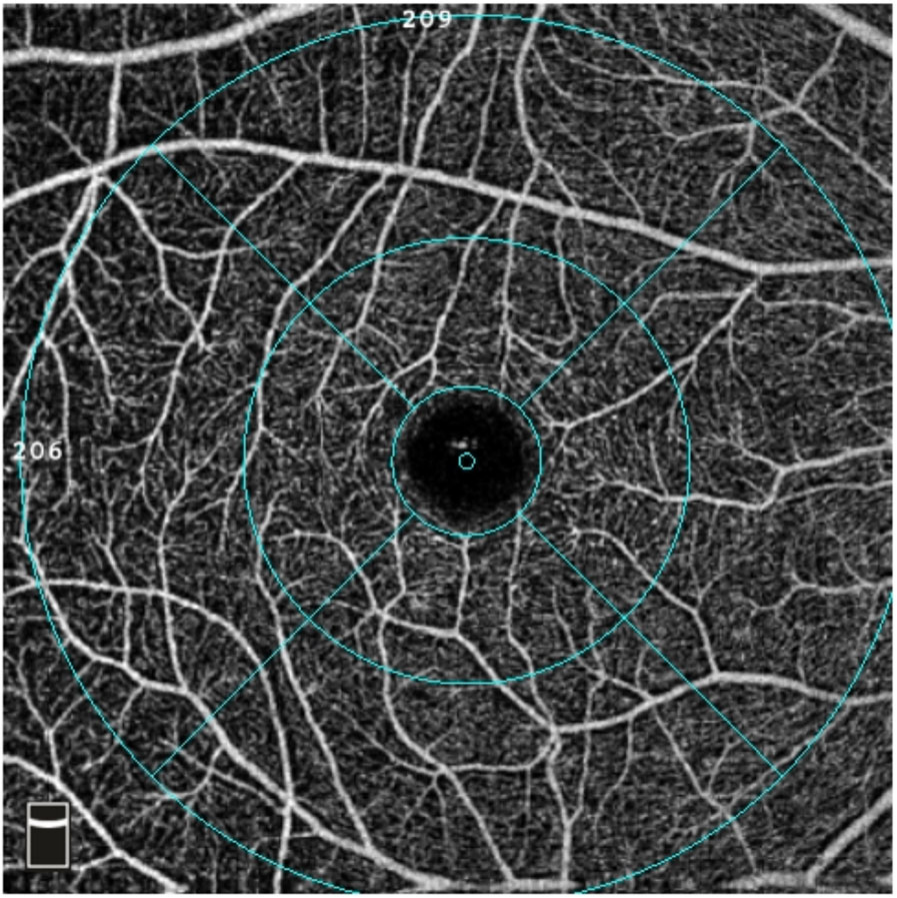
Macular vessel density was automatically labeled as the fovea, parafoveal and perifoveal region

We selected the macular HD 6 × 6 mm program with a scanning area centered on macula to evaluate macular microcirculation. The image was automatically divided into the superficial capillary plexuses (SCP) and deep capillary plexuses (DCP). Seen in Fig. 1. SCP is defined as a lamina extending from internal limiting membrane (ILM) to 10 μm above inner plexiform layer (IPL) while DCP is the lamina extending from 10 μm above IPL to 10 μm below outer plexiform layer (OPL). Vessel density (%) was quantified automatically by the inner software of OCT system. Besides, the software automatically fits a circle (1.0 mm in diameter) centered on the fovea. The parafovea region is defined as a 2.0 mm wide round annulus around the fovea and the perifovea region is defined as a 3.0 mm wide round annulus around the parafovea. Seen in Fig. 2.

**Fig. 3:**
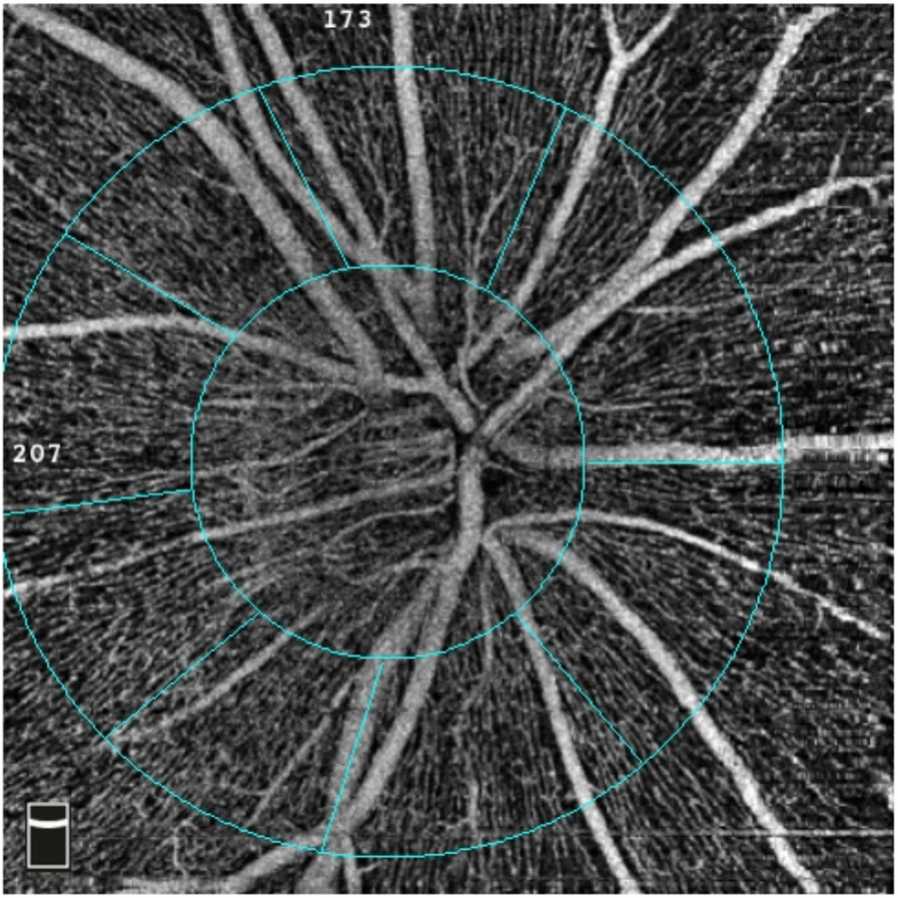
Example of an optic nerve head vessel density image. The software automatically fits the optic disc region and peripapillary region

To evaluate blood flow of optic nerve head (ONH), a HD disc 4.5 × 4.5 mm program was acquired and vessel density (%) was quantified automatically by the OCT system. The software automatically fits a circle (2 mm in diameter) centered on the optic disc and the peripapillary region is defined as a 1.0 mm wide round annulus around the optic disc 2.0 mm circle [10]. Seen in Fig. 3.

Poor quality images with a scan quality less than 7 were excluded from analysis. Therefore, data of 3 of our patients were excluded.

### Statistical analysis

Qualified data were processed and analyzed with SPSS for Mac software (Version 25.0; SPSS, Chicago, IL). All data were normal distributed confirmed by a Kolmogorov-Smirnoff test. Measurements before surgery was compared with measurements 1 day/1 week/1 month after surgery respectively using the repeated-measures analysis. *P* < 0.05 was considered statistically significant.

## Results

The study included 45 eyes of 45 individuals who received FS-LASIK in our hospital. There were 15 male and 30 female patients with average age of 27.1 ± 4.44 years (range: 21 to 40 years). Preoperative spherical equivalent were − 5.66 ± 2.18 D (range: − 8.25D to − 1.50D) with a spherical refraction of − 5.31 ± 2.15D (range: − 8.00D to − 1.50D) and a cylindrical refraction of − 0.82 ± 0.69D (range: − 2.25D to 0.50D). The duration of suction ring application in surgery were 51.75 ± 14.4 s (range: 38 to 93 s). Seen in Table. 1.

**Table 1:** Baseline characteristics

Characteristics	Mean ± Standard Deviation
Age	27.1 ± 4.44 years
Gender	15 males: 30 females
Manifest refraction (D)	
Sphere	−5.31 ± 2.15D
Cylinder	−0.82 ± 0.69D
Spherical equivalent	−5.66 ± 2.18 D
Suction time	51.75 ± 14.4 s

Vessel density (VD) of whole 6 × 6 mm macular image in the SCP and DCP were 50.84 ± 0.4% and 52.95 ± 0.74% respectively before surgery. The VD of whole macular image in SCP decreased after surgery, but the changes were not significant (F(3,132) = 1.41, *P* = 0.24). VD of the whole macular image at DCP showed significant decrease 1 day and 1 week after surgery (49.64 ± 0.81%, *P* = 0.001 at 1 day, 50.46 ± 0.91%, *P* = 0.022 at 1 week) and returned to its preoperative level 1 month after surgery (51.4 ± 0.81%, *P* = 0.102). Measurements of the VD of foveal, parafoveal and perifoveal regions in SCP and DCP were shown in Table 2 and Table 3.

**Table 2:** Vessel density (VD) (Mean ± SD) of macular superficial plexus at baseline and 1 day/1 week/1 month after FS-LASIK

	Baseline (mean ± SD) %	1 day (mean ± SD) %	1 week (mean ± SD) %	1 month (mean ± SD) %
Whole area (6*6mm^2^)	50.84 ± 0.4	49.98 ± 0.39	50.06 ± 0.49	50.42 ± 0.47
Fovea Region	21.66 ± 1.01	20.03 ± 1.01^*^	19.97 ± 0.97^*^	20.06 ± 0.94^*^
Parafovea Region	53.31 ± 0.54	51.73 ± 0.49	51.89 ± 0.67	52.54 ± 0.6
Perifovea Region	51.56 ± 0.41	50.77 ± 0.43	50.76 ± 0.49	51.09 ± 0.49

VD at baseline was compared with VD at 1 day/1 week/1 month respectively using the repeated-measures analysis. The VD of whole area of SPC had no significantly change after surgery (F(3,132) = 1.41, *P* = 0.24). The preoperative VD of fovea region was significantly difference from the VD at 1 day/1 week/1 month after surgery (F = (2.71,118.83) = 9.72, *P* < 0.001). VD at 1 day/1 week/1 month after surgery decreased significantly (*P* < 0.001). VD of parafovea and perifovea region did not change postoperatively (F(3,132) = 2.27,*P* = 0.08; F(3,132) = 1.23,*P* = 0.301, respectively). * represented *P* < 0.05

**Table 3:** Vessel density (VD) (Mean ± SD) of macular deep plexus at baseline and 1 day/1 week/1 month after FS-LASIK

	Baseline (mean ± SD) %	1 day (mean ± SD) %	1 week (mean ± SD) %	1 month (mean ± SD) %
Whole area (6*6mm^2^)	52.95 ± 0.74	49.64 ± 0.81^*^	50.46 ± 0.91^*^	51.4 ± 0.81
Fovea Region	38.22 ± 1.17	36.14 ± 1.19^*^	36.21 ± 1.2^*^	36.35 ± 1.17^*^
Parafovea Region	58.27 ± 0.49	55.56 ± 0.56^*^	55.91 ± 0.8^*^	56.93 ± 0.54^*^
Perifovea Region	54.14 ± 0.83	50.59 ± 0.91^*^	51.67 ± 1.01^*^	52.63 ± 0.9

VD at baseline was compared with VD at 1 day/1 week/1 month respectively using the repeated-measures analysis. The VD of whole area of DPC significantly differ after surgery (F(3,132) = 4.45, *P* = 0.005). Vessel density was decreased 1 day after surgery (*P* = 0.001, *P* = 0.022 at 1 week and 1 month respectively) and gradually recovered to its normal range in 1 month (*P* = 0.102). The preoperative VD of fovea region was significantly difference from the VD at 1 day/1 week/1 month after surgery (F = (3132) = 11.73, *P* < 0.001). VD at 1 day/1 week/1 month after surgery decreased significantly (*P* < 0.001). VD of parafovea region significantly changed after surgery (F(2.71,119.26) = 5.13,*P* = 0.003). Vessel density of parafovea region was decreased after surgery (*P* < 0.001, *P* = 0.01, *P* = 0.039 at 1 day/1 week/1 month respectively). The perifovea region also changed postoperatively (F(3,132) = 4.09,*P* = 0.008). the vessel density was decreased at 1 day (*P* = 0.001) and gradually returned to its preoperatively level in 1 month (*P* = 0.04, *P* = 0.142 at 1 week and 1 month respectively). * represented *P* < 0.05

**Fig. 4:**
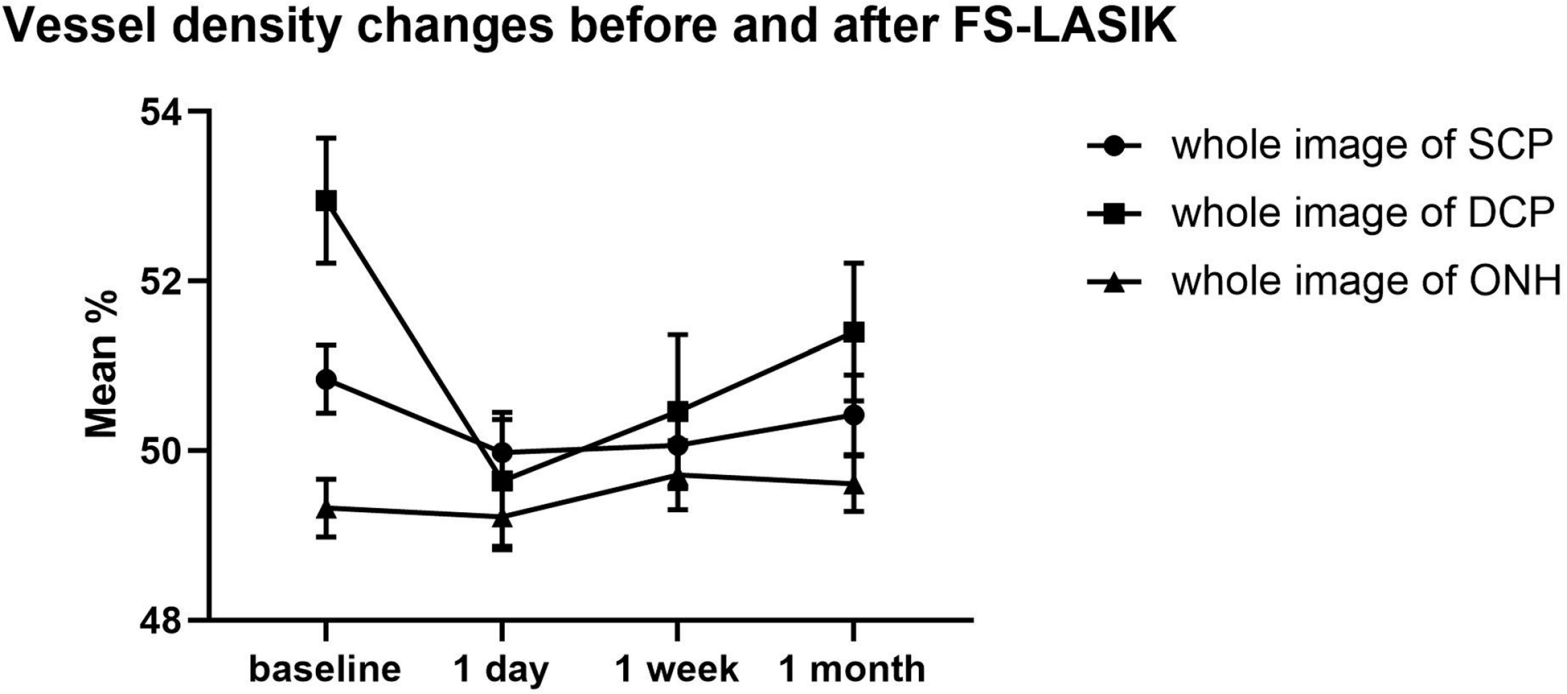
Tendency of vessel density changes at the whole images of macular superficial capillary plexus (SCP), deep capillary plexus (DCP) and ONH (optic nerve head) before and after surgery

VD in the whole 4.5 × 4.5 mm ONH region showed no significant change before and after surgery (F(2.51,95.18) = 0.6, *P* = 0.588). However, a decrease in VD values inside the disc was observed 1 day after surgery (*P* = 0.003), and the vessel density returned to its preoperative level 1 week after surgery (*P* = 0.098). VD in the peripapillary area did not change significantly after the FS-LASIK (F(2.93,90.75) = 1.44, *P* = 0.24). (Table 4.) The tendency of vessel density in whole image of SCP, DCP and ONH was shown in Fig. 4.

**Table 4:** Vessel density (VD) (Mean ± SD) of optic nerve head region at baseline and 1 day/1 week/1 month after FS-LASIK

	Baseline (mean ± SD) %	1 day (mean ± SD) %	1 week (mean ± SD) %	1 month (mean ± SD) %
Whole area (4.5*4.5mm^2^)	49.32 ± 0.34	49.22 ± 0.35	49.17 ± 0.41	49.61 ± 0.33
Inside disc region	58.51 ± 0.52	56.6 ± 0.65	57.09 ± 0.84	58.61 ± 0.67
Peripapillary Region	51.16 ± 0.42	51.91 ± 0.42	51.76 ± 0.43	51.88 ± 0.35

VD at baseline was compared with VD at 1 day/1 week/1 month respectively using the repeated-measures analysis. VD of whole area (F(2.51,95.18) = 0.6, *P* = 0.588), inside disc region (F(2.32,88) = 5.38, *P* = 0.08), peripapillary region (F(2.39,90.75) = 1.44, *P* = 0.24) of ONH did not differ after surgery

## Discussion

By modifying the central corneal curvature, LASIK is frequently used for correcting refractive errors. Before the first clinical use of femtosecond laser for LASIK flap creation in 2001 [11], the corneal flap was created by a mechanical microkeratome. Femtosecond laser has a high laser frequency, which permits lower energy per pulse and tighter line separation, thereby creating a smoother corneal flap and resulting in faster visual recovery [12–14]. Besides, the rapid development of femtosecond (10^− 15^ s) lasers over the past two decades has opened up new applications in ophthalmic surgery [12] such as FLEx (FS laser lenticular extraction), SMILE (small-incision lenticular extraction), FLACS (FS laser-assisted cataract surgery), etc. Many studies have revealed significant IOP increases during FS laser-assisted procedures [15–17]. In spite of changes in intraocular pressure, a phenomenon called retinal autoregulation [18–21] is able to maintain blood flow and oxygen supply to the fundus at a constant level. Rats experiments have [22] observed that as the intraocular pressure increased by 30 mmHg to 100 mmHg, autoregulation was overwhelmed, and the retinal blood flow diminished linearly. Maximum IOP during corneal flap cutting with a femtosecond laser was reported vary from 27mmHg [23] to 328mmHg [16] by different researches. To our knowledge, this is the first study to investigate the retinal microcirculation changes associated with transient IOP spike caused by FS-LASIK in human.

In the present study, we found that macular vessel density of whole image at DCP descreased significantly in early recovery period after FS-LASIK, but VD returned to its preoperative value 1 month after surgery. However, VD of macular subregions at SCP and DCP showed significant change 1 month after surgery especially the foveal region at both layers and the parafoveal region at DCP (Table 2.). FS-LASIK did not significantly change vessel density at the whole ONH region.

Ozdamar et al. observed a temporary increase in blood flow at the lamina cribrosa region (choroidal blood flow) of the ONH by using Heidelberg Retinal Flowmeter, suggesting a compensatory change for maintaining blood flow in the ONH following Lasik-induced ischemia [9]. While Miriam et al. found that all blood-flow responses returned immediately to normal levels [3], including the systemic blood-flow and ocular blood-flow measured by Doppler imaging system. However, Doppler scanning system is insensitive to microcirculation, and visualization has typically been limited to major veins and arteries [24]. OCTA utilizes a Split-Spectrum Amplitude-Decorrelation Angiography (SSADA) algorithm which is more sensitive to microcirculation of the retina. In our study, a sudden change of IOP during corneal flap cutting did not affect the microcirculation in ONH region, which matched the results found by Miriam et al. compared with the mechanical microkeratome, femtosecond laser system employs a lower pressure suction ring to stabilize the globe [12] could be another reason why it did not affect the microcirculation in ONH region.

Previous study of blood flow during LASIK microkeratome ring found no blood-flow signal in the central retinal artery during the suction phase [4]. Usually, the only arterial blood supply to the inner retina is from the central retinal artery [25]. The artery divides to trunks and then branches to supply oxygen and nutrition to the inner retina. And the retinal venous branches are distributed in a relatively similar pattern. Branches from the central retinal vessels dive deep into the retina forming two distinct capillary beds, the superficial capillary plexus and the deep capillary plexus. The morphology and function of the retinal capillary layers are coupled with the metabolic demands of the neuronal retina. Animal model experiments [26, 27] have found that the dominant oxygen consumers of the inner retina are located in the plexiform layers, which are supplied by the DCP. This may explain the reason why macular VD at DCP decreased at the early period after surgery. The VD in DCP is more sensitive to the oxygen alterations caused by a sudden increase of IOP during surgery. Both the overall macular VD in SCP and DCP had no changes 1 month after surgery, suggesting that the sudden increase of IOP during femtosecond laser procedure in LASIK did not cause a long-term change of microcirculation in macular region. However, significant decrease of VD in fovea at both SCP and DCP and significant decrease of VD in parafoveal region at DCP were observed 1 day after surgery and remained significantly declined 1 month after surgery. Previous studies have found that LASIK for myopia significantly deteriorated contrast sensitivity and it took at least 6 months for recovery of contrast sensitivity (CS) [28]. It is known that change of the foveal microstructure may affect deterioration or improvement of CS [29]. The decrease of VD in fovea and parafoveal region may explain the deterioration of CS after LASIK. The relation between changes in macular vessel density and contrast sensitivity would be important in our future studies.

Limitations of this study include that the real-time IOP was unable to be measured because of an aseptic requirement in surgery and a limited space to operate measurement during the femtosecond procedure. Second, we did not perform OCTA immediately after surgery because the intense tearing and itching will affect the quality of images. Third, we could have added a control group of patients scheduled for LASEK, which a suction phase was not necessary during the surgery. But the regeneration of corneal epithelium after LASEK will affect the image quality of OCTA in early recovery. Last, a sufficiently lengthy period of follow-up observation would ensure that there would be no long-term damage to the microcirculation in macular and ONH region.

## Conclusion

In conclusion, the sudden increase of IOP in FS-LASIK caused a temporary decrease of vessel density at the deep layer of macular region. Vessel density at optic nerve head region had no significant changes after surgery. Transient IOP spike during FS-LASIK did not cause long-term decline of retinal microcirculation after surgery. However, vessel density at the fovea and parafovea region of deep capillary plexus were declined and did not recover to their preoperative levels 1 month after surgery. Further investigation with a longer follow-up period will be needed to confirm no long-term damage would remain at the fovea and parafoveal region.

## Data Availability

The datasets used and analyzed during the current study are available from the corresponding author on reasonable request.

## Abbreviations

CS: Contrast sensitivity
DCP: Deep capillary plexuses
FS-LASIK: Femtosecond laser assisted Laser in situ keratomileusis
OCTA: Optical coherence tomography angiography
ONH: Optic nerve head
SCP: Superficial capillary plexuses
VD: Vessel density
